# Association between tuberculosis, diabetes and 25 hydroxyvitamin D in Tanzania: a longitudinal case control study

**DOI:** 10.1186/s12879-016-1960-x

**Published:** 2016-11-03

**Authors:** Noémie Boillat-Blanco, Pascal Bovet, Kaushik L. Ramaiya, Maliwasa Mganga, Lilian T. Minja, Lanja Saleh, Medea Imboden, Christian Schindler, Sebastien Gagneux, Claudia Daubenberger, Klaus Reither, Nicole Probst-Hensch

**Affiliations:** 1 Ifakara Health Institute, Dar es Salaam, United Republic of Tanzania; 2 Swiss Tropical and Public Health Institute, Basel, Switzerland; 3 Department of Sciences, University of Basel, Basel, Switzerland; 4 Infectious Diseases Service, Lausanne University Hospital, Lausanne, Switzerland; 5 Institute of Social and Preventive Medicine, Lausanne University Hospital, Lausanne, Switzerland; 6 Shree Hindu Mandal Hospital and Muhimbili University of Health Sciences, Dar es Salaam, United Republic of Tanzania; 7 Kinondoni Municipal Council, National Tuberculosis Program, Dar es Salaam, United Republic of Tanzania; 8 Institute of Clinical Chemistry, University of Zurich, University Hospital of Zurich, Zurich, Switzerland

**Keywords:** Stress-induced hyperglycemia, Transient hyperglycemia, Tuberculosis, Vitamin D, Diabetes

## Abstract

**Background:**

Vitamin D level is inversely associated with tuberculosis (TB) and diabetes (DM). Vitamin D could be a mediator in the association between TB and DM. We examined the associations between vitamin D, TB and DM.

**Methods:**

Consecutive adults with TB and sex- and age-matched volunteers were included in a case-control study in Dar es Salaam, Tanzania. Glycemia and total vitamin D (25(OH)D) were measured at enrolment and after TB treatment in cases. The association between low 25(OH)D (<75 nmol/l) and TB was evaluated by logistic regression adjusted for age, sex, body mass index, socioeconomic status, sunshine hours, HIV and an interaction between low 25(OH)D and hyperglycemia.

**Results:**

The prevalence of low 25(OH)D was similar in TB patients and controls (25.8 % versus 31.0 %; *p* = 0.22). In the subgroup of patients with persistent hyperglycemia (i.e. likely true diabetic patients), the proportion of patients with low 25(OH)D tended to be greater in TB patients (50 % versus 29.7 %; *p* = 0.20). The effect modification by persistent hyperglycemia persisted in the multivariate analysis (p_interaction_ = 0.01).

**Conclusions:**

Low 25(OH)D may increase TB risk in patients with underlying DM. Trials should examine if this association is causal and whether adjunct vitamin D therapy is beneficial in this population.

**Electronic supplementary material:**

The online version of this article (doi:10.1186/s12879-016-1960-x) contains supplementary material, which is available to authorized users.

## Background

Diabetes mellitus (DM) triples the risk of tuberculosis (TB) and the global increase of DM in developing countries is expected to have an important impact on TB incidence [1, 2]. Several studies have shown an association between low blood levels of vitamin D with both TB and DM [3–5]. Vitamin D is mainly generated in the skin exposed to sunlight and, to a lower extent, through dietary intake [6]. Vitamin D is usually measured as total blood level of 25 hydroxyvitamin D (25(OH)D) which represents vitamin D bound to albumin and vitamin D binding proteins (VDBP) as well as free vitamin D and does not represent only the levels of the free, active metabolite (1,25(OH)_2_D) which requires conversion from 25(OH)D. The free biological active vitamin D, 1,25(OH)_2_D, has a short half-life and, therefore, 25(OH)D measurement is more representative of the vitamin D status of an individual. However, the level of the free active vitamin D can be modifiedby the level of binding proteins as well as by the binding affinity of VDBP which is affected by genotype [7].

Vitamin D deficiency was first described as the cause of rickets and osteomalacia. However, the vitamin D receptor is expressed in most cells in the body and vitamin D seems to have an effect on a number of other health outcomes [6]. The association between type 2 DM and low vitamin D has been described in several cross-sectional and prospective studies and a meta-analysis of 21 prospective studies showed an inverse association between blood levels of 25(OH)D and risk of type 2 DM, particularly when 25(OH)D was <50 nmol/l [8]. Vitamin D affects pancreatic β-cell function, boosts the antimicrobial activity of human macrophages against *Mycobacterium tuberculosis*, and modulates adaptive response [9]. Building on these findings, it was suggested that a low blood level of vitamin D could mediate some of the association between DM and TB [10]. A cross-sectional Indian study confirmed the association between severe vitamin D deficiency (<25 nmol/l), TB and DM while another study conducted in China did not [11, 12].

A trial showed that vitamin D supplementation accelerates the resolution of inflammatory responses during TB treatment and may be used as a host-directed therapy [13]. However, the role of adjunctive vitamin D on TB outcome remains uncertain as several randomized clinical trials of vitamin D supplementation did not show a beneficial effect on TB outcome or mortality [14–18]. Vitamin D supplementation might have a role in the prevention and treatment of TB in targeted subgroups of patients with low vitamin D levels, such as diabetic patients.

In this study, we aimed to describe first, the determinants of low vitamin D level among healthy controls and TB patients and, second, the association between TB, vitamin D, and the presence and persistence of hyperglycemia in an African equatorial population.

## Methods

### Study design and setting

This case-control study with longitudinal follow-up of cases was part of a study on the association between TB and DM [19]. In this sub-study, TB patients were recruited between February and December 2013 and followed up for a median time of 5.8 months (IQR 4.7–9.9) after the start of anti-TB treatment. Controls were recruited between March and September 2013 and did not have follow-up visits. Glucose and 25(OH)D were measured in blood collected at baseline from cases (88 % before onset of TB treatment) and controls, and in blood collected at follow-up from cases (54 % still under TB treatment). The study was carried out in Kinondoni, the most populated District of Dar es Salaam. Patients were recruited in Mwananyamala Regional Hospital and connected health facilities (Sinza Hospital, Magomeni Health Centre, Tandale Dispensary). Dar es Salaam is located at a latitude of −6.81° and longitude of 39.28° which ensures a good ability of sunlight to synthesize vitamin D [6].

### Study participants

#### Cases

Consecutive adult patients (age ≥18 years; living in Kinondoni District) presenting in the participating hospitals with new active TB diagnosed by the National TB and Leprosy Control Programme (NTLP) were screened for study inclusion. TB diagnosis was based on sputum smear microscopy, chest X-ray (read by an experienced radiologist), clinical evidence of TB and decision by the clinician to treat with a full-course of anti-TB therapy [20]. TB patients were classified as pulmonary smear-positive, smear-negative or extrapulmonary according to NTLP guidelines [21]. TB patients were treated for 6 months with a standard regimen or longer if necessary [20, 22].

#### Controls

We used frequency matching on sex and age (10-year age groups) to select controls among adults accompanying patients (other than the one included in the study) to the outpatient departments and living in Kinondoni District. Exclusion criteria were a biological relationship to case, TB history, symptoms or signs of TB, other acute infection or major trauma within the last 3 months.

### Study procedures

Demographic characteristics, DM history and symptoms, socioeconomic status (SES; indicators of education, occupation, wealth using factor analysis), smoking (ever daily smoking), and alcohol misuse (≥three drinks per day or ≥ six drinks per occasion) were obtained. Daily sunshine hours’ data were provided by the Tanzanian meteorological agency. Data were entered directly into an open data kit in a personal digital assistant with real-time error, range and consistency checks [23]. Participants were screened for HIV infection according to the national algorithm (rapid immune-chromatographic test (Alere Determine™ HIV-1/2) confirmed by a second rapid test (Trinity Biotech Uni-gold™ Recombigen® HIV-1/2)).

#### Total 25 hydroxyvitamin D measurement

Serum was kept at −80 °C within 6 h of blood draw and blood 25(OH)D level was assessed by electrochemoluminescence immunoassay (Cobas® 8000, Roche Diagnostics) in the Institute of Clinical Chemistry at the University Hospital of Zurich in Switzerland. Low blood vitamin D status was defined as a 25(OH)D <75 nmol/l and vitamin D deficiency as a 25(OH)D <50 nmol/l [6, 24]. Of note, no patient received vitamin D replacement therapy.

#### Hyperglycemia screening

Blood glucose testing was conducted after an overnight fast of ≥8 h (fasting capillary glucose, FCG) and 2-h glucose in an oral glucose tolerance test (2-hCG; standard 75-g OGTT; GlucoPlus™, Diabcare, plasma-calibrated glucometer which accuracy conformed to the International Standardization Organization guidelines) [25]. Abnormal glycemic results (FCG ≥5.6 mmol/l or 2-hCG ≥7.8 mmol/l) were confirmed by repeat testing 2 to 5 days later. For safety reasons, 2-hCG testing was omitted if FCG was ≥11.1 mmol/l and DM was diagnosed. Hyperglycemia refers to patients with repeated FCG ≥6.1 mmol/l and/or 2-hCG ≥7.8 mmol/l according to WHO [26]. Persistent hyperglycemia was defined based on hyperglycemia measured at enrolment and confirmed at follow-up and is considered a proxy for pre-existing DM. Hyperglycemia detected at enrolment only is more likely to reflect stress hyperglycemia as a result of TB [19]. Participants diagnosed with DM (FCG >7 mmol/l or 2-hCG >11 mmol/l) were referred to the local DM clinic, but none was started on DM treatment. Indeed, they all had a fasting glycemia below 10 mmol/l and it is the usual practice in Tanzania not to treat these patients.

### Data analysis

Characteristics of TB patients were compared to controls. Factors associated with low 25(OH)D were identified by comparison with participants with a normal level. Differences were tested with Wilcoxon-Mann-Whitney and chi-square tests. The independent association between low 25(OH)D and TB, as well as between persistent hyperglycemia (suggestive of underlying DM) and TB, was evaluated by multivariate logistic regression. We also assessed the interaction between low 25(OH)D and persistent hyperglycemia (suggestive of underlying DM) to examine if the association between TB and vitamin D might differ according to underlying DM status.

To assess the combined effect of low 25(OH)D and hyperglycemia, a 4-level categorical variable reflecting the combination of underlying DM and vitamin D status was used as predictor variable in the logistic regression models. Covariates included age, sex, body mass index (BMI), SES, mean daily sunshine hours during the month of enrolment, HIV status.

Statistical analyses were performed using Stata software (StataCorp, College Station, TX, USA, version 12) and GraphPad Prism 6. *P* values <0.05 were considered as significant.

## Results

### Study sample

**Fig. 1:**
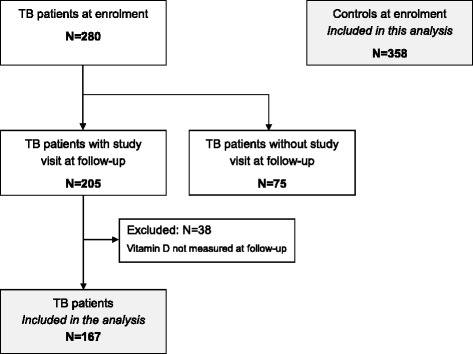
Flow chart of study participants. Abbreviation: TB: tuberculosis

**Fig. 2:**
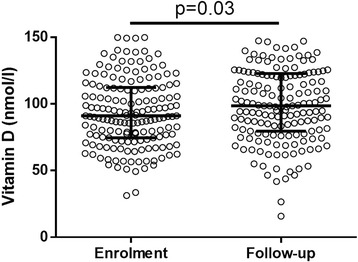
Categorical scatter plot representing the longitudinal evolution of vitamin D level between baseline and follow-up. Bars represent the 25^th^ percentiles, the median and the 75^th^ percentiles. The difference between enrolment and follow-up was tested with Wilcoxon-Mann-Whitney test. Abbreviation: TB: tuberculosis

At enrolment, 280 TB patients and 358 controls had blood sampled for 25(OH)D and glucose measurement. The final study sample consisted of 167 TB cases with follow-up information and 358 controls, all of whom had complete information on glycemic status, 25(OH)D and relevant covariates (Fig. 1). Characteristics of the study sample are described in Table 1. Compared to controls, TB patients were more often HIV-infected, previously known for DM and had a lower BMI. At enrolment, hyperglycemia was more common in TB patients than in controls. There was no longer a difference when looking at persistent hyperglycemia, i.e. excluding patients with stress hyperglycemia secondary to TB infection [19]. Among TB patients with hyperglycemia at enrolment, 34 % (*N* = 13) had a FCG and/or 2-hCG in the DM range and 68 % had a normal glycemia after TB treatment although none of them received treatment for DM and only three had lifestyle counselling (Additional file 1: Figure S1). Blood 25(OH)D increased during TB treatment (mean ± SD increase 6.5 ± 21 nmol/, *p* = 0.03) independently of ongoing TB treatment at the time of measurement (Fig. 2). Correspondingly, the prevalence of low 25(OH)D decreased (25.8 % versus 20.4 %, *p* < 0.001).

**Table 1:** Characteristics at baseline of the case-control sample

	TB patients *N* = 167	Healthy controls *N* = 358	
	N (%) or Mean (sd)		*p*
Age	33.7 (10.7)	36.1 (13.0)	0.05
Male sex	95 (56.9)	191 (53.4)	0.45
History of smoking	25 (15.0)	90 (25.3)	0.008
Alcohol misuse	16 (9.6)	22 (6.2)	0.16
Socioeconomic status			
Low	41 (24.6)	71 (19.9)	0.25
Medium	82 (49.1)	185 (52.0)	0.64
High	44 (26.4)	100 (28.1)	0.75
Mean daily sunshine hours during the month of enrolment	7.9 (1.4)	7.9 (1.1)	0.08
Body Mass Index (kg/m^2^)	22.5 (4.2)	25.1 (5.1)	<0.001
HIV infection	51 (30.7)	51 (14.3)	<0.001
Previously known DM	5 (3.0)	2 (0.6)	0.02
Hyperglycemia at enrolment	38 (22.8)	37 (10.3)	<0.001
Persistent hyperglycemia	12 (7.2)	37 (10.3)	0.33
25 hydroxyvitamin D deficiency	3 (1.8)	21 (5.9)	0.04
Low 25 hydroxyvitamin D	43 (25.8)	111 (31.0)	0.22
25 hydroxyvitamin D level (nmol/l)	94.0 (26.9)	89.6 (26.9)	0.08
TB characteristics			
TB symptoms >3 months	21 (12.6)		
TB			
Smear positive	136 (81.4)		
Smear negative	27 (16.2)		
Extrapulmonary	4 (2.4)		
Cavity on X-ray	86 (52.8)		

Abbreviations and definitions: *TB* tuberculosis, *Alcohol misuse* ≥3 drinks per day or ≥6 drinks per occasion, *Socioeconomic status* assessed with indicators of scholar education, occupation and wealth ownership using factor analysis, *Persistent hyperglycemia* presence of hyperglycemia at enrolment confirmed at follow up (measure of glycemia repeated among patients with tuberculosis only), *Low vitamin D* <75 nmol/l, *Vitamin D deficiency* <50 nmol/l. *Hyperglycemia *fasting capillary glucose >6 mmol/l and/or 2-hCG >7.7 mmol/l, *TB symptoms >3months *>3months duration of tuberculosis symptoms.

*P* values were calculated using the Wilcoxon-Mann-Whitney test for continuous variables and chi-square tests for categorical variables

Characteristics and glycemic and vitamin D status of TB patients with and without follow-up were comparable, except for underweight and male sex being more common among subjects without follow-up (Additional file 2: Table S1).

### Correlates of low 25(OH)D level

In the control group, low 25(OH)D was more common in women than in men, but less common in HIV infected persons. In TB patients, low 25(OH)D was associated with underlying DM (assessed as either a previous DM diagnosis or as persistent hyperglycemia at enrolment and at follow up; Table 2). Level of 25(OH)D was not associated with TB phenotype, but the analysis was limited by a low number of TB patients with extrapulmonary disease.

**Table 2:** Factors associated with low 25 hydroxyvitamin D level among healthy controls and tuberculosis patients at enrolment

	TB patients (*N* = 167)			Healthy controls (*N* = 358)		
	Low 25(OH)D level	Normal 25(OH)D level		Low 25(OH)D level	Normal 25(OH)D level	
	*N* = 43 (25.7 %)	*N* = 124 (74.3 %)		*N* = 111 (31.0 %)	*N* = 247 (69.0 %)	
	N (%) or Mean (sd)	N (%) or Mean (sd)	*p*	N (%) or Mean (sd)	N (%) or Mean (sd)	*p*
Age	35.1 (12.0)	33.3 (10.2)	0.53	35.5 (12.4)	36.4 (13.3)	0.79
Male sex	20 (46.5)	75 (60.5)	0.11	46 (41.4)	144 (58.8)	0.002
History of smoking	4 (9.3)	21 (16.9)	0.23	25 (22.5)	65 (26.8)	0.40
Alcohol misuse	0 (0)	7 (5.7)	0.11	3 (2.7)	19 (7.7)	0.07
Socioeconomic status						
Low	11 (25.6)	30 (24.2)	0.84	17 (15.3)	54 (22.2)	0.2
Medium	22 (51.2)	60 (48.4)	0.86	57 (51.4)	127 (52.3)	1
High	10 (23.3)	34 (27.4)	0.84	37 (33.3)	62 (25.5)	0.13
Mean daily sunshine hours during the month of enrolment	8.2 (1.2)	7.9 (1.4)	0.26	7.9 (1.0)	7.9 (1.1)	0.22
Body mass index (kg/m^2^)	19.8 (3.1)	20.4 (3.9)	0.54	25.8 (5.5)	24.9 (4.8)	0.11
HIV infection	12 (27.9)	39 (31.7)	0.64	6 (5.4)	45 (18.4)	0.001
Previously known for DM	4 (9.3)	1 (0.8)	0.005	0 (0)	2 (0.8)	0.34
Hyperglycemia at enrolment	9 (20.9)	29 (23.4)	0.74	11 (9.9)	26 (10.5)	0.86
Persistent hyperglycemia	6 (14.0)	6 (4.8)	0.05			
TB characteristics						
TB symptoms >3 months	8 (18.6)	13 (10.5)	0.17			
TB						
Smear positive	36 (83.7)	100 (80.7)	0.82			
Smear negative	6 (14.0)	21 (16.9)	1			
Extrapulmonary	1 (2.3)	3 (2.4)	1			
Cavity on X-ray	20 (48.8)	66 (54.1)	0.56			

Abbreviations and definitions: *25(OH)D* 25 hydroxyvitamin D, *Low vitamin D level* <75 nmol/l, *Alcohol misuse* ≥3 drinks per day or ≥6 drinks per occasion, *Socioeconomic status* assessed with indicators of scholar education, occupation and wealth ownership using factor analysis, *DM* diabetes, *Persistent hyperglycemia* presence of hyperglycemia at enrolment and at follow up (measure of glycemia repeated among patients with tuberculosis only). *Hyperglycemia *fasting capillary glucose >6 mmol/l and/or 2-hCG >7.7 mmol/l, *TB symptoms >3months* >3 months duration of tuberculosis symptoms before diagnosis.

*P* values were calculated using the Wilcoxon-Mann-Whitney test for continuous variables and chi-square tests for categorical variables

### Association between TB and low 25(OH)D in all subjects and according to persistent hyperglycemia

**Fig. 3:**
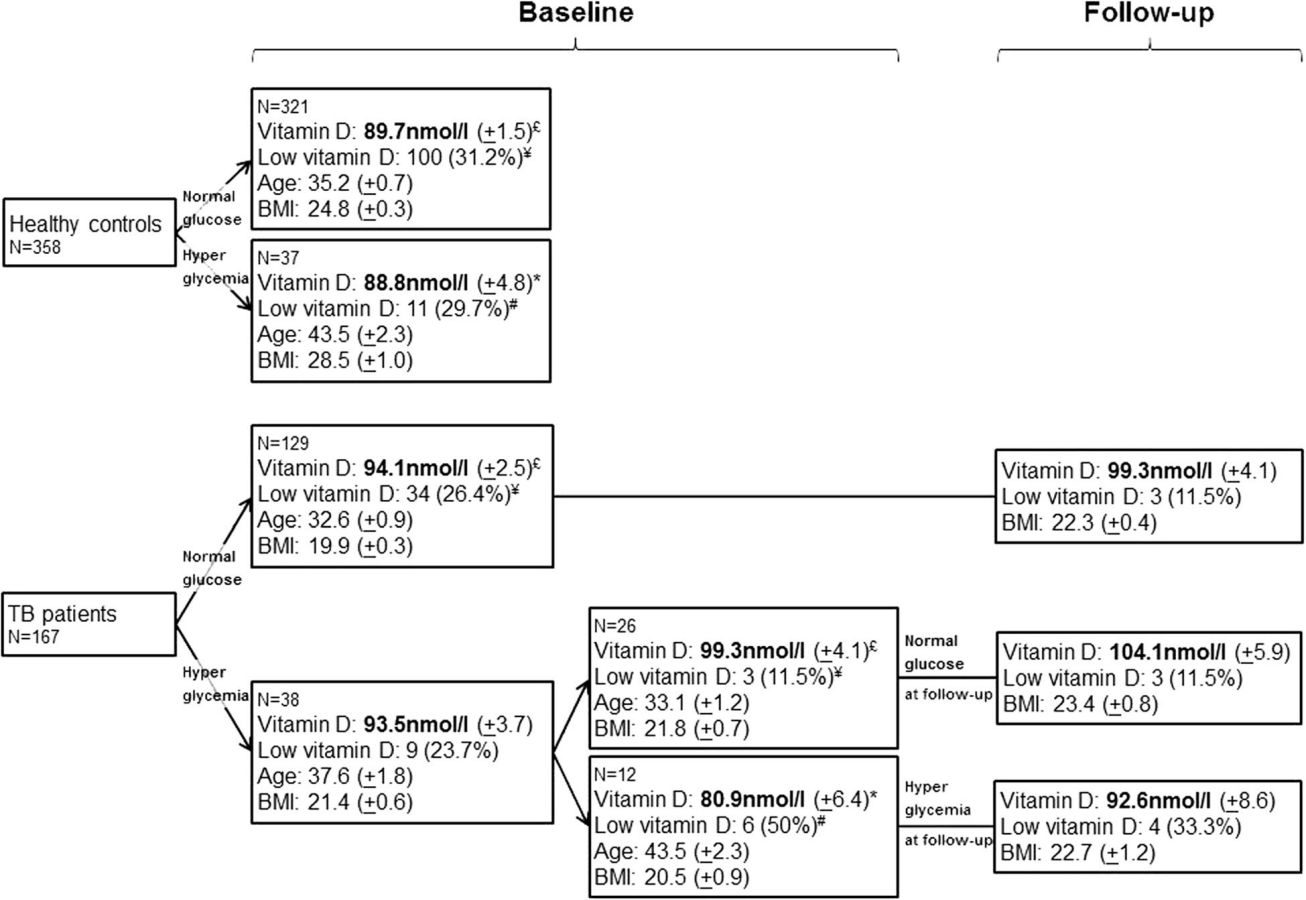
Longitudinal evolution of vitamin D level and selected variables according to tuberculosis status and hyperglycemia at baseline and follow-up. Abbreviation: TB: tuberculosis. 25(OH)D concentration and 25(OH)D status were compared according to TB status after stratification by persistent hyperglycemia using *P* values calculated by the Wilcoxon-Mann-Whitney test for continuous variables and chi-square tests for categorical variables. * *p* = 0.33; # *p* = 0.20; £ *p* = 0.05; ¥ *p* = 0.11

Overall, the proportion of patients with a low 25(OH)D was not statistically different in TB patients compared to controls (25.8 % versus 31.0 %; *p* = 0.22). In the subgroup of participants with persistent hyperglycemia (i.e. likely true diabetic patients), baseline 25(OH)D tended to be lower in TB patients than in controls (mean (±SE) 80.9 nmol/l ±6.4 versus 88.8 nmol/l ± 4.8; *p* = 0.33) and the proportion of patients with low baseline 25(OH)D tended to be greater (50 % versus 29.7 %; *p* = 0.20; Fig. 3). In other words, unadjusted analysis suggests an association of low 25(OH)D with TB status among participants with persistent hyperglycemia.

In contrast, in patients without persistent hyperglycemia, 25(OH)D was higher in TB patients than in controls, irrespective of the time point of vitamin D measurement (mean (±SE) 95.0 (±2.2) versus 89.7 (±1.5); *p* = 0.05) and the proportion of patients with low 25(OH)D tended to be lower (23.9 % versus 31.2 %; *p* = 0.10).

Neither low 25(OH)D nor persistent hyperglycemia were associated with an increased risk of TB when occurring in isolation. However, the combination of the two factors was associated with a fourfold increase in the odds of TB compared to patients without the two risk factors. While this difference was not quite statistically significant (*p* = 0.08), the interaction of the two factors was (adjusted OR (95 % CI): 12.42 (1.78−86.60); p_interaction_ = 0.01; Table 3). Additional adjustment for the month of recruitment had no effect on the results presented in Table 3.

**Table 3:** Association with tuberculosis in different patients’ categories according to vitamin D and glycemic status

	Association with tuberculosis	
	Adjusted OR (95 % CI)^a^	*P* value
Normal 25 hydroxyvitamin D level and absence of persistent hyperglycemia	*Ref.*	*Ref.*
Low 25 hydroxyvitamin D level and absence of persistent hyperglycemia	0.70 (0.41–1.20)	0.20
Normal 25 hydroxyvitamin D level and persistent hyperglycemia	0.46 (0.15–1.44)	0.18
Low 25 hydroxyvitamin D level and persistent hyperglycemia	4.0 (0.86–18.54)	0.08

Interaction factor between low 25 hydroxyvitamin D and persistent hyperglycemia: adjusted OR (95%CI) 12.42 (1.78–86.60); p_interaction_ = 0.01

^a^Adjusted for age, sex, body mass index, socioeconomic status, mean daily sunshine hours during the month of enrolment and HIV status.

*Low vitamin D level* <75 nmol/l, *Persistent hyperglycemia* presence of hyperglycemia at enrolment confirmed at follow up (measure of glycemia repeated among patients with tuberculosis only).

Adjusted odds ratios and *p* values were calculated using multivariate logistic regression

The interaction was not significant when using the continuous value of 25(OH)D (adjusted OR (95 % CI): 0.97 (0.93−1.01); p_interaction_ = 0.09).

## Discussion

In this black sub-Saharan equatorial population, we provide novel evidence for the association between low 25(OH)D and TB among persons with persistent hyperglycemia, irrespective of HIV status. In contrast, 25(OH)D levels were higher in TB cases compared to controls in normoglycemic participants. The longitudinal design of this study reinforces the validity of our findings as it makes possible to differentiate between persons with only transient hyperglycemia possibly related to the acute phase of inflammation versus participants with persistent hyperglycemia who are likely truly diabetic patients. ().

The associations between low vitamin D status and DM, low vitamin D status and TB as well as TB and DM have been demonstrated in many studies. These data point to vitamin D as a potential mediator in the link between DM and TB which is consistent with our results [10]. Our data are also concordant with an Indian study showing that the prevalence of severe vitamin D insufficiency (<25 nmol/L) was higher among TB patients with DM compared to those without DM [11]. However, in our study, the definition of low vitamin D was different (<75 nmol/L) as most participants had a high 25(OH)D concentration. The absence of an association between DM and 25(OH)D among healthy controls may be explained by the low statistical power related to the low numbers of diabetic patients. Our longitudinal study underlines the limitations of studying the combined impact of circulating glucose and 25(OH)D on TB in a cross-sectional manner only (i.e. based on data at baseline only). At the time of diagnosis of active TB, the inflammation status can influence hyperglycemia (stress-induced hyperglycemia) and likely also 25(OH)D levels (e.g. through induction of 25(OH)D metabolism or transient changes in the blood levels of proteins bound to 25(OH)D) [19, 27, 28]. Hence, cross-sectional studies may miss the importance of DM (persistent hyperglycemia) in the association between TB and vitamin D status [19]. Consistent with two other studies, 25(OH)D level increased during TB treatment in our study, despite the described lowering effect of isoniazid and rifampicin on blood vitamin D [29–31]. The improved vitamin D status during TB treatment may be related to greater sun exposure and dietary intake of vitamin D during recovery. However, results related to 25(OH)D have to be interpreted carefully, as 25(OH)D mainly reflects protein-bound vitamin D and may not represent the level of free active form of vitamin D. During inflammation, similarly to levels of several other blood acute-phase proteins, the concentration of albumin decreases and might modify 25(OH)D result [27], while one study showed no change in blood VDBP during the first 8 weeks of TB treatment [28]. Further studies should examine the relation between TB, diabetes and vitamin D based on levels of free vitamin D.

The importance of low blood levels of vitamin D as a risk factor for the development of TB was suggested in a prospective cohort study conducted among HIV-infected adults in Tanzania [32]. Several case control studies also reported a lower blood 25(OH)D in TB patients compared to control groups [33, 34]. However, consistent with a large study conducted in Mwanza, Tanzania, we observed an increased 25(OH)D among TB patients compared to healthy controls, particularly after treatment of TB [29]. Also, contrary to many studies elsewhere, mean 25(OH)D was in the normal range in the Tanzanian studies, possibly because of higher vitamin D levels in a tropical country with high exposure to UVB compared to countries remote of the Equator, which may have limited the potential impact of low 25(OH)D on TB to only the group of diabetic patients who had lower 25(OH)D levels [35]. Indeed, vitamin D deficiency varies between studies conducted in Africa, ranging from 4.3 % in Tanzania to 62.7 % in South Africa [33].

Our study has several strengths. First, the longitudinal design allowed assessing the association between TB, DM and vitamin D after resolution of inflammation, which influences glycemic status. Indeed, many TB patients with hyperglycemia at baseline have a normal glycemia after TB treatment which suggests that many cross-sectional studies on the association between TB and DM probably overestimate the increased TB risk associated with DM. The study population was well defined and the patients categorized according to WHO recommendations. Our study also has limitations. First, we performed the measurement of total 25(OH)D and not its active form (1,25(OH)_2_D) [7]. TB diagnosis was based on NTLP guidelines and smear results rather than culture or GenXpert. However, the study was conducted under the real case scenario of TB treatment in this population. Fasting and 2-h glucose were assessed on capillary whole blood using a point-of-care test and not in venous blood. However, we used a plasma-calibrated glucometer the accuracy of which conformed to the International Standardization Organization guidelines [25]. Another limitation of our study is that healthy controls did not have a follow-up assessment of their glycemic status. However, we did not expect a temporal modification of DM status over 5 months’ follow-up in the general population as we included only participants who did not present any acute infection or major trauma within the last 3 months. We used persistent hyperglycemia as a proxy of DM in TB patients to exclude patients with stress hyperglycemia secondary to TB infection. This definition may have missed some DM patients who had improvement of their glycemic status by changing their lifestyle during the follow-up period. However, very few patients (*N* = 3) went to the DM clinic and had lifestyle counselling and it is unlikely that it significantly contributed to our results. Vitamin D deficiency was recently identified as a probable risk factor for extrapulmonary TB [36]. However, the number of TB patients with this phenotype was very low in our study and did not allow any subgroup analysis. The impact of 25(OH)D level on TB outcome is controversial and adjunctive vitamin D may improve TB outcome in subgroups of patients only [15, 18, 31, 37]. Unfortunately, we did not have the power to look at the effect of the interaction between low 25(OH)D and persistent hyperglycemia on TB outcome.

## Conclusions

In this equatorial population with a high mean 25(OH)D concentration (91 nmol/L), a low blood level of 25(OH)D (<75 nmol/L) seems to increase the risk of TB only in the context of persistent hyperglycemia. Diabetic patients might be an appropriate target for vitamin D supplementation to improve TB outcome or prevent active TB, but the benefit of such interventions requires confirmation through randomized controlled trials.

## Additional files

Additional file 1:
**Figure S1.** Longitudinal evolution of glycemic status among tuberculosis patients between enrolment (left bar) and follow up (right bar). Abbreviation: pre-DM: pre-diabetes, defined as fasting capillary glucose between 6.1 and 7 mmol/l and/or 2-h capillary glucose between 7.8 and 11.0 mmol/l; DM: diabetes, defined as fasting capillary glucose >7 mmol/l and/or 2-h capillary glucose >11.0 mmol/l, according to WHO recommendation. (JPG 46 kb)
Additional file 2:
**Table S1.** Characteristics of the TB patients with and without follow-up. (DOCX 15 kb)

## Abbreviations

1,25(OH)_2_D: 1,25-Dihydroxyvitamin D
2h-CG: 2-h capillary glucose after standard oral glucose tolerance test
25(OH)D: 25-hydroxyvitamin D
95 % CI: 95 % Confidence interval
aOR: Adjusted odds ratio
BMI: Body mass index
DM: Diabetes mellitus
FCG: Fasting capillary glucose
NTLP: National tuberculosis and leprosy control programme
OGTT: Oral glucose tolerance testing
pre-DM: Pre-diabetes
SD: Standard deviation
SES: Socio-economic status
TB: Tuberculosis
WHO: World Health Organization
