# Supplementary figures and images for: Association between tuberculosis, diabetes and 25 hydroxyvitamin D in Tanzania: a longitudinal case control study
Source: BMC Infect Dis. 2016 Nov 3;16:626. doi: 10.1186/s12879-016-1960-x (PMC5096317; doi:10.1186/s12879-016-1960-x)

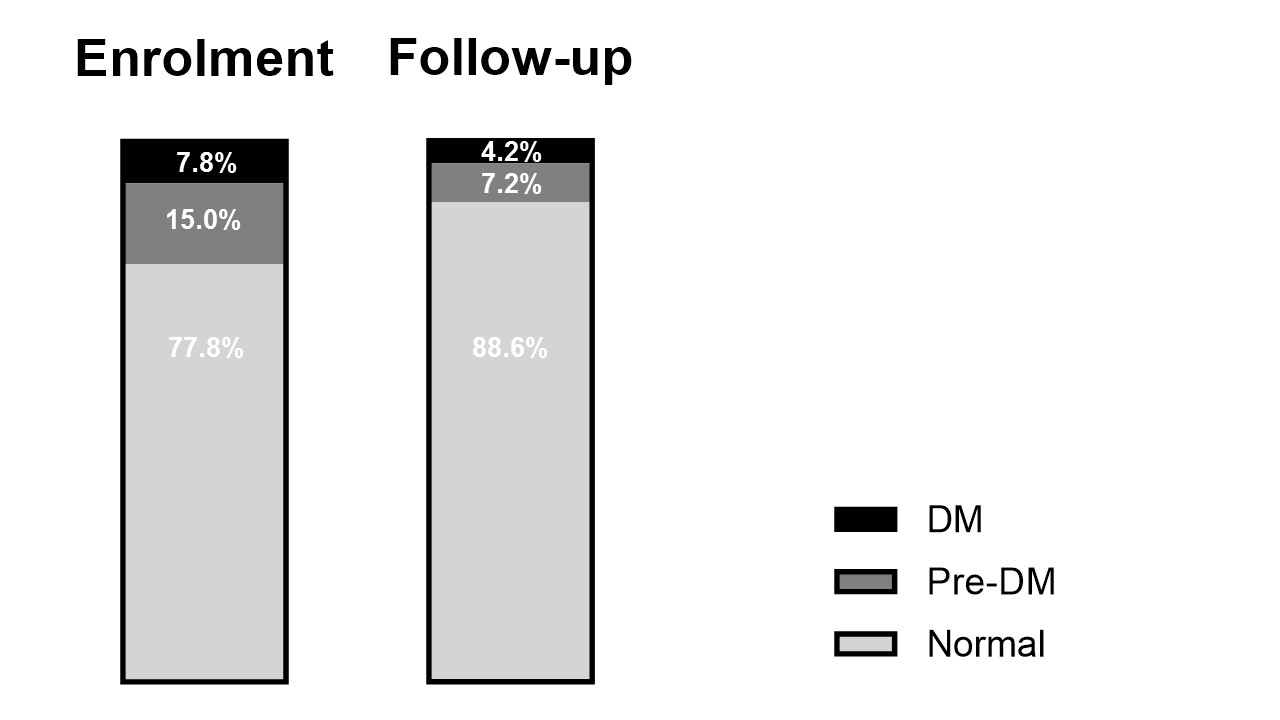

Supplement: Additional file 1: — Figure S1. Longitudinal evolution of glycemic status among tuberculosis patients between enrolment (left bar) and follow up (right bar). Abbreviation: pre-DM: pre-diabetes, defined as fasting capillary glucose between 6.1 and 7 mmol/l and/or 2-h capillary glucose between 7.8 and 11.0 mmol/l; DM: diabetes, defined as fasting capillary glucose >7 mmol/l and/or 2-h capillary glucose >11.0 mmol/l, according to WHO recommendation. (JPG 46 kb) [file 12879_2016_1960_MOESM1_ESM.jpg]
