# Supplementary material for: Association between tuberculosis, diabetes and 25 hydroxyvitamin D in Tanzania: a longitudinal case control study
Source: BMC Infect Dis. 2016 Nov 3;16:626. doi: 10.1186/s12879-016-1960-x (PMC5096317; doi:10.1186/s12879-016-1960-x)
Supplement: Additional file 2: — Table S1. Characteristics of the TB patients with and without follow-up. (DOCX 15 kb) [file 12879_2016_1960_MOESM2_ESM.docx]

**Table S1.** Characteristics of the TB patients with and without follow-up.

|  | All TB patients |  | TB patients with all data available | TB patients without follow-up data available |  |  |
| --- | --- | --- | --- | --- | --- | --- |
|  | N=280 |  | N=167 (59.6) | N=113 (40.4) |  |  |
|  | N(%) or Mean(sd) |  | N(%) or Mean(sd) | | p |  |
|  |  |  |  |  |  |  |
| Age |  |  | 33.7 (10.7) | 34.2 (11.2) | 0.83 |  |
| Male sex |  |  | 95 (56.9) | 82 (72.6) | 0.008 |  |
| History of smoking |  |  | 25 (15.0) | 30 (26.6) | 0.02 |  |
| Alcohol Misuse |  |  | 7 (4.2) | 14 (12.4) | 0.01 |  |
| Socioeconomic status |  |  |  |  | 0.26 |  |
| Low |  |  | 41 (24.6) | 37 (32.7) |  |  |
| Medium |  |  | 82 (49.1) | 53 (46.9) |  |  |
| High |  |  | 44 (26.4) | 23 (20.4) |  |  |
| Sunshine exposure (daily hours) |  |  | 7.9 (1.4) | 7.9 (1.3) | 0.83 |  |
| Body Mass Index (kg/m^2^) |  |  | 20.2 (3.7) | 19.4 (3.9) | 0.04 | |
| HIV infection |  |  | 51 (30.7) | 29 (25.9) | 0.38 | |
| Hyperglycemia |  |  | 38 (22.8) | 25 (22.1) | 0.90 | |
| Previously known DM |  |  | 5 (3.0) | 1 (0.9) | 0.23 | |
| Low vitamin D |  |  | 43 (25.8) | 26 (23.0) | 0.60 | |
| Vitamin D (nmol/l) |  |  | 94.0 (26.9) | 95.0 (32.1) | 0.59 | |
| TB symptoms >3M |  |  | 21 (12.6) | 17 (15.0) | 0.55 | |
| TB |  |  |  |  | 0.42 | |
| Smear positive |  |  | 136 (81.4) | 98 (86.7) |  | |
| Smear negative |  |  | 27 (16.2) | 12 (10.6) |  | |
| Extrapulmonary |  |  | 4 (2.4) | 3 (2.7) |  | |
| Cavity on X-ray |  |  | 86 (52.8) | 65 (61.3) | 0.17 | |
|  |  |  |  |  |  | |

Abbreviations and definitions: Alcohol misuse: ≥weekly alcohol consumption / socioeconomic status: assessed with indicators of scholar education, occupation and wealth ownership using factor analysis / Hyperglycemia: fasting capillary glucose: ≥6.1 mmol/l and/or 2-hour capillary glucose: ≥7.8 mmol/l

P values were calculated using the Wilcoxon_Mann_Whitney test for continuous variables and chi-square tests for categorical variables.
